# High dose oral and intravenous rifampicin for improved survival from adult tuberculous meningitis: a phase II open-label randomised controlled trial (the RifT study)

**DOI:** 10.12688/wellcomeopenres.14691.1

**Published:** 2018-07-10

**Authors:** Fiona V. Cresswell, Kenneth Ssebambulidde, Daniel Grint, Lindsey te Brake, Abdul Musabire, Rachel R. Atherton, Lillian Tugume, Conrad Muzoora, Robert Lukande, Mohammed Lamorde, Rob Aarnoutse, David Meya, David R. Boulware, Alison M. Elliott

**Affiliations:** 1 Clinical Research Department, London School of Hygiene and Tropical Medicine, London, WC1E 7HT, UK; 2 Clinical Research, Infectious Diseases Institute, Kampala, Uganda; 3 Tropical Epidemiology Group, London School of Hygiene and Tropical Medicine, London, WC1E 7HT, UK; 4 Department of Pharmacy, Radboud University Medical Centre, Nijmegan, Netherlands; 5 Mbarara University of Science and Technology, Mbarara, Uganda; 6 Department of Pathology, College of Health Sciences, Makerere University, Kampala, Uganda; 7 Division of Infectious Diseases, University of Minnesota, Minneapolis, USA; 8 MRC - UVRI - LSHTM Uganda Research Unit, Entebbe, Uganda

**Keywords:** TBM, Tuberculous Meningitis, TB, rifampicin, Ultra, HIV

## Abstract

**Background**: Tuberculous meningitis (TBM) has 44% (95%CI 35-52%) in-hospital mortality with standard therapy in Uganda. Rifampicin, the cornerstone of TB therapy, has 70% oral bioavailability and ~10-20% cerebrospinal fluid (CSF) penetration.  With current WHO-recommended TB treatment containing 8-12mg/kg rifampicin, CSF rifampicin exposures frequently fall below the minimal inhibitory concentration for *M. tuberculosis*. Two Indonesian phase II studies, the first investigating intravenous rifampicin 600mg and the second oral rifampicin ~30mg/kg, found the interventions were safe and resulted in significantly increased CSF rifampicin exposures and a reduction in 6-month mortality in the investigational arms. Whether such improvements can be replicated in an HIV-positive population remains to be determined.

**Protocol**: We will perform a phase II, open-label randomised controlled trial, comparing higher-dose oral and intravenous rifampicin with current standard of care in a predominantly HIV-positive population. Participants will be allocated to one of three parallel arms (I:I:I): (i) intravenous rifampicin 20mg/kg for 2-weeks followed by oral rifampicin 35mg/kg for 6-weeks; (ii) oral rifampicin 35mg/kg for 8-weeks; (iii) standard of care, oral rifampicin 10mg/kg/day for 8-weeks. Primary endpoints will be: (i) pharmacokinetic parameters in plasma and CSF; (ii) safety. We will also examine the effect of higher-dose rifampicin on survival time, neurological outcomes and incidence of immune reconstitution inflammatory syndrome. We will enrol 60 adults with suspected TBM, from two hospitals in Uganda, with follow-up to 6 months post-enrolment.

**Discussion**: HIV co-infection affects the bioavailability of rifampicin in the initial days of therapy, risk of drug toxicity and drug interactions, and ultimately mortality from TBM. Our study aims to demonstrate, in a predominantly HIV-positive population, the safety and pharmacokinetic superiority of one or both investigational arms compared to current standard of care. The most favourable dose may ultimately be taken forward into an adequately powered phase III trial.

**Trial registration**: ISRCTN42218549 (24 ^th^ April 2018)

## Introduction

### Background

Worldwide, 10.4 million people are estimated to have fallen ill with Tuberculosis (TB) in 2016, resulting in 1.3 million deaths, and an additional 374,000 TB deaths in people living with HIV ^1^. The African region carries the greatest burden relative to its population; 281 cases for every 100,000 people, more than double the global average of 133 per 100,000. Additionally, of the 1.2 million HIV-infected people who developed TB worldwide, 74% were in the African World Health Organisation (WHO) region ^2^.

The true incidence of TB meningitis is not known due to lack of accurate diagnostics and scarce epidemiological data in many TB endemic regions. Central nervous system (CNS) TB accounts for ~1% of notified TB cases in Germany and the USA, but this proportion is likely to be significantly higher in HIV endemic regions where TB frequently manifests as disseminated disease ^3, 4^. As such, in the wake of the HIV epidemic; TB meningitis (TBM) is one of the leading causes of meningitis in both adults and children in Africa ^5, 6^. It is estimated there is likely to be well over 100,000 cases annually worldwide.

### Prognosis of TBM

TBM, the most devastating form of TB, is associated with a mortality of 7–28% of HIV-uninfected ^7^, and 29–63% of HIV-infected patients ^8, 9^. The driver of this 2 to 3-fold increased risk of death in HIV co-infection has not yet been elucidated. Neuro-disability is seen in up to 50% of survivors, regardless of their HIV serostatus. TBM cases account for up to 19% of all hospitalized HIV-associated TB cases and pose a significant challenge to healthcare services and care-givers, often requiring prolonged hospital stays and long rehabilitation periods ^10^. Delays in seeking medical care, diagnosis, and initiation of treatment are contributing factors to the high morbidity and mortality, particularly in resource-limited settings.

### Clinical TBM management

Early treatment with anti-tuberculous therapy and adjunctive corticosteroids is the most effective way of reducing death and disability from TBM. However, currently morbidity and mortality remains unacceptably high ^5^. Whilst the 4 ^th^ edition of the WHO treatment guidelines suggest substituting ethambutol with streptomycin during the intensive phase of treatment this has not been widely adopted for a number of reasons, and treatment for TBM does not differ from that of pulmonary TB, with rifampicin (R), isoniazid (H), pyrazinamide (Z) and ethambutol (E) given for two months (intensive phase) followed by rifampicin and isoniazid (continuation phase), except that continuation phase is prolonged to complete 9–12 months ^2, 11^.

Due to variable TB drug penetration across the blood-brain barrier and blood-cerebrospinal fluid (CSF) barrier, optimal treatment regimens for pulmonary TB may not be the most effective options for TBM. Inadequate CNS drug penetration may be an important contributory factor to the high early mortality in TBM ^12^. Improved early treatment outcomes may be achieved by altering drug selection, drug doses, and routes of administration to ensure adequate drug delivery to the site of disease and maximal early mycobactericidal activity in the CNS.

### Rifampicin in TBM

Rifampicin is the cornerstone drug in the treatment of TBM, as evidenced by the fact that those infected with rifampicin-resistant *Mycobacterium tuberculosis* ( *M.tb*) strains have a near-universal fatal outcome, even with treatment with second-line drugs in resource-rich settings ^5, 13, 14^. Mortality is not nearly so high in patients with isoniazid mono-resistant TBM ^6^.

In addition to male sex and low body weight, which are associated with more rapid drug clearance ^15^, malabsorption may contribute to reduced systemic drug bioavailability in HIV-infected persons ^16– 21^. A recent comprehensive meta-analysis of rifampicin pharmacokinetics confirmed that during the initial days of TB treatment rifampicin total plasma exposure to rifampicin is reduced in HIV co-infected adults (AUC 37.2mg.h/L in HIV-positive versus 56.7mg.h/L in HIV-negative, p=0.003). Interestingly, this association did not persist in steady-state (>7 days on therapy, once saturation of first-pass metabolism and the establishment of metabolic autoinduction is well established) ^22^. Lower plasma levels of antituberculous agents are predictive of poorer outcomes in pulmonary TB ^23^, thus the lower plasma levels of rifampicin seen in HIV-positive individuals in the initial days of TBM therapy may be particularly relevant in TB meningitis, which carries a high early mortality. Specific factors that potentially play a role in reduced bioavailability include HIV-related enteric infections ^17, 18^, HIV-associated enteropathy and chronic intestinal immune activation affecting regulation of drug transporters. Patients with TBM often present while critically ill, are vomiting and frequently receive drugs through a nasogastric tube. Such factors may further associate with reduced TB drug concentrations, which could explain the lower plasma rifampicin concentrations observed in patients with TBM compared to those with pulmonary TB taking the same dose from the same setting ^24, 25^.

Rifampicin is largely protein bound and thus has limited CSF penetration, with reported CSF concentrations often reaching 10–20% of those in plasma ^25, 26^. Suggested target concentrations of rifampicin are >8mcg/ml at the site of disease, but CSF rifampicin concentrations seldom exceeded the minimum inhibitory concentration (MIC; 0.25mcg/ml) against *M. tuberculosis* at the standard oral adult dose (8–12mg/kg) ^12^. Currently, little is known about rifampicin concentrations in brain matter, spinal cord or meninges and how plasma levels or inflammation affect tissue concentrations.

The evidence for the currently recommended dose of rifampicin (10mg/kg/day) in TBM treatment is scant, and this dose falls on the steep part of the dose-response curve for sterilising effect ^27^. Recent studies by the Pan-African Consortium for the Evaluation of Antituberculous Antibiotics in pulmonary TB have shown that rifampicin dosed at 35mg/kg is associated with improved early bactericidal activity and 2-fold faster rate of sputum conversion (Hazard Ratio 1.99, 95% CI:1.21-3.29) as compared to standard therapy. Furthermore, this dose was safe and well tolerated, with a 14% grade 3-5 adverse event incidence as compared with 10% with standard 4-drug TB therapy with rifampicin dosed at 10mg/kg ^28^.

Ruslami *et al*. investigated the safety and pharmacokinetic (PK) profiles of higher than normal dose *intravenous* (IV) rifampicin as well as oral moxifloxacin in adults with TBM. Although not powered to detect a mortality benefit, improved survival was observed in patients receiving 600mg IV rifampicin (~13mg/kg/day) compared to the oral standard dose (~10mg/kg/day). Patients who received IV rifampicin had a more rapid resolution of coma (4 versus 5 days), reduced mortality at 8-weeks (24% versus 55%) and 6-months (34% versus 65%); (adjusted Hazard Ratio=0.42; 95% CI 0.20-0.91, p=0.03) compared with those who received oral standard dose rifampicin. The change in rifampicin dose and mode of delivery was associated with a 3-fold increase in plasma area under the time-concentration curve (AUC) and maximum concentration (C _max_) and a 3-fold increase in CSF C _max_ from 0.21 to 0.60 mcg/mL.

A second phase II study in Indonesia evaluating increased *oral* doses of 30mg/kg or 20mg/kg versus standard of care (10mg/kg) also reported no increase in toxicity in the high dose arms and CSF C _max_ was significantly increased (25.5 mg/L [11.9 to 55.5], 18.1 mg/L [2 to 43.6], 7.2 mg/L [2.2 to 14.1] respectively, <0.001). In those with microbiologically confirmed TBM there was a trend towards lower 6-month mortality in the 30mg/kg arm compared to standard of care (7% versus 36% (HR 0.16 [0.02 to 1.34], p=0.09) ^29^.

In contrast, a large Vietnamese trial of an intensified TBM treatment containing oral rifampicin 15mg/kg/day versus standard of care showed no differential effect on mortality (27.7% versus 27.9%, hazard ratio 0.94; 95%CI 0.73 to 1.22; p=0.66) ^30^

The conflicting evidence from prior trials evaluating the benefits of higher than standard doses of rifampicin for TBM creates a situation of equipoise that can only be answered with an adequately powered phase III trial examining a rifampicin dose with proven pharmacokinetic-pharmacodynamic superiority to the standard of care. This phase II study aims to demonstrate pharmacokinetic superiority of one or both of the investigational arms so that the most favourable dose may ultimately be taken forward into a phase III study. Additionally, it is important to generate PK and safety data from HIV-positive African adults, as HIV co-infection can have a significant impact on TBM outcomes, pharmacokinetics and drug toxicity.

## Protocol

This is version 1.2.1, 19 ^th^ February 2018.

### Hypotheses

Our **primary hypothesis** is that intravenous rifampicin (20mg/kg) and high dose oral rifampicin (35mg/kg) will result in significantly increased plasma and CSF exposure during the critical early days of TBM treatment as compared to standard control (10mg/kg oral rifampicin).

Our **secondary hypotheses** are that high dose rifampicin will lead to improved early mycobacterial clearance from the CNS, reduced inflammatory response, and thereby will result in more rapid resolution of coma, improved long-term functional status, reduced TBM immune reconstitution inflammatory syndrome (IRIS) incidence and lower mortality.

### Main study objectives

***Primary objective***. Our primary objective is to determine whether higher-dose rifampicin, delivered either orally at 35mg/kg/day or intravenously at 20 mg/kg/day for 2-weeks (followed by orally at 35 mg/kg/day for 6-weeks) is safe and provides exposure profiles that are favourable compared to the 10 mg/kg standard dose oral rifampicin.

***Secondary objectives***. Our secondary objectives are to observe whether greater rifampicin exposure in CSF is associated with any clinical benefit including more rapid resolution of coma, improved long-term functional status, reduced TBM-IRIS incidence and lower mortality.

### Ancillary studies

Two ancillary studies will be conducted within the RifT trial, the second of which will only take place at Mulago Hospital.

***Evaluation of the diagnostic accuracy of Xpert MTB/Rif Ultra (Ultra) in TBM***. Our hypothesis is that CSF Ultra is significantly more sensitive than CSF Xpert MTB/Rif or culture in the diagnosis of TBM.

***Descriptive study of brain tissue rifampicin concentrations on autopsy specimens***. Our hypothesis is that brain tissue concentrations will correlate with plasma and CSF concentration and will be significantly higher in the investigational arms.

### Design and setting

**Figure 1.**
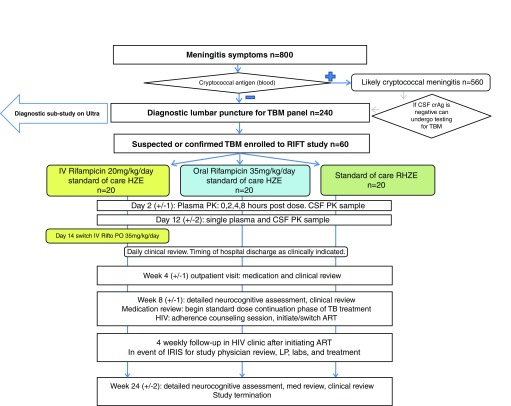
Study flow chart.

RifT is a three arm, parallel group, phase II open label randomised controlled trial (see Figure 1), evaluating three rifampicin regimens over an 8-week intervention period, as follows:

A. Intravenous 20mg/kg/day rifampicin for 2-weeks (followed by oral rifampicin 35mg/kg/day for 6-weeks)
B. Oral 35mg/kg/day rifampicin for 8-weeks
C. Standard of care oral rifampicin (~10mg/kg/day) for 8-weeks

All participants will receive a standard backbone of oral TB treatment (consisting of isoniazid ~5mg/kg/day, pyrazinamide ~25mg/kg/day, ethambutol ~20mg/kg/day) and daily IV dexamethasone (0.4mg/kg/day) for 1 week then weaned over a 6–8 week period, as per WHO guidelines.

The trial will be set in two hospitals in Uganda: Mulago National Referral Hospital and Mbarara Regional Referral Hospital. The study population will be adults (≥18 years of age) with a diagnosis of TBM and treatment for TBM planned by the attending physician.

### Endpoints

***Primary end points***

*1. Pharmacokinetic parameters*

Plasma and CSF rifampicin area under the curve (AUC _0-24h_), and maximum concentration (C _max_)

*2. Adverse events*

We will use a composite safety endpoint for the 8-week intervention period that includes clinical grade 3-5 adverse events (AEs) (as classified by Division of AIDS (DAIDS) Toxicity Scale) or all serious adverse events (SAEs), drug-induced liver injury (DILI), grade 3-5 defined by alanine transaminase (ALT) >5x upper limit of normal (ULN), or discontinuation of rifampicin for >5 consecutive days in the first 8 weeks for any cause.

***Secondary endpoints***

*1. Mortality*

Mortality at 8- and 24-weeks post randomisation will be compared between study arms.

*2. Time to normalization of conscious level*

We will determine days from randomisation until observation of a GCS of 15 which is achieved for ≥2 consecutive days.

*3. Functional status*

The modified Rankin scale at 8 and 24 weeks will be used in determining functional outcomes.

*4. TB immune reconstitution inflammatory syndrome*

TB-IRIS will be diagnosed according to published case definitions ^31^. TB-IRIS will be determined independently by 2 trial clinicians blinded to treatment allocation.

### Inclusion and exclusion criteria

***Inclusion criteria***. Participants to be included in the study are consecutive patients ≥18 years with clinically suspected TBM based on meningitis symptoms, clinical signs of meningism and anti-tuberculous chemotherapy planned by the attending physician. In addition, they must have either a bedside CSF glucose to plasma ratio <50%, or absolute CSF glucose <40mg/dl or 2.2mmol/l or a positive CSF acid fast bacilli (AFB) smear or positive Xpert MTB/Rif or Ultra. Written informed consent must be given by either by the participant or by surrogate if the participant has altered mental state.

***Exclusion criteria***. Patients with jaundice or known liver cirrhosis will not be eligible to enrol. Additionally, those who have received more than 3 doses of TB treatment within the previous 3 days, or have discontinued TB treatment in the prior 14 days, or those with known allergy to rifamycins, isoniazid, pyrazinamide, ethambutol or study drug excipients are not eligible. Those with known current/previous rifampicin drug-resistant *M. tuberculosis* infection or those with concurrent cryptococcal meningitis or those known to be currently taking any drug that has a clinically relevant interaction with rifampicin or other first-line TB drugs, including ritonavir, atazanavir or darunavir will not be eligible. Individuals who cannot or are unlikely to attend regular clinic visits or are pregnant or breastfeeding or where we lack consent from participant or family members are not eligible. Those with known porphyria or known chronic renal failure with eGFR <10ml/min are not eligible.

### Randomisation and treatment allocation

Adults who have given consent for, and undergone, a diagnostic screening lumbar puncture and are eligible for enrolment will then be approached for enrolment consent after which they will be randomised. Randomisation will occur at study entry and must occur prior to the 4th dose of TB treatment. Participants will be randomised by a computer generated permutated block randomization algorithm of different sized blocks with a 1:1:1 randomization ratio into the three arms described above.

Randomisation will be stratified by:

1. Clinical site
2. British Medical Research Council (BMRC) TBM disease grade I or II/III at time of consent

All randomised participants will initiate their allocated study TB treatment within 24 hours of randomisation, and preferably the same day. As TBM is a medical emergency, initiation of standard of care TB treatment will not be delayed whilst waiting for randomisation.

***Treatment discontinuation***. Due to the urgent need to begin TBM therapy to reduce mortality, enrolment will not be delayed whilst waiting for enrolment blood test results and participants will be replaced *a posteriori* if baseline ALT>3x ULN. As infection with a rifampicin resistant TB strain is an exclusion criterion, all patients in whom rifampicin resistant TB is identified after randomisation may have their treatment altered as per physician judgement. Once randomised, analysis is by modified intent-to-treat (ITT).

### Interventions

***Diagnostic lumbar puncture***. At the time of screening, after informed consent has been received, a diagnostic lumbar puncture will be performed with a ~10ml CSF sample collected. The sample with then be tested with an enhanced TB diagnostic panel including Xpert MTB/Rif, Ultra, MGIT culture, Biofire meningoencephalitis multiplex polymerase chain reaction and next generation sequencing.

***Antituberculous therapy***

**Intravenous rifampicin arm (R20IV)**

For the first 14 days study drug will be administered as intravenous rifampicin 20mg/kg once daily. Study medications will be dispensed during hospitalization to ward staff who will administer the drugs to the participants under directly observed therapy. Rifampicin 600mg will be reconstituted with 10 ml of sterile water for a dilution of 60 mg per ml of reconstituted solution. The weight-specific volume of rifampicin solution will be drawn up ( Table 1) then further diluted in 500 ml 5% Dextrose solution. The solution will be administered as a continuous infusion via a peripheral venous catheter over 2 hours. Thereafter the line will be flushed with 5 ml 0.9% normal saline after which the cannula will be capped. Other antituberculous drugs (isoniazid, pyrazinamide and ethambutol) will be given orally by weight ( Table 2) as intact tablets unless they are unable to swallow voluntarily, e.g. in participants with a depressed level of consciousness, in which case tablets will be crushed and given via nasogastric tube.

**Table 1.** Intravenous administration of rifampicin by weight for day 0–14 in the intravenous rifampicin arm (arm R20IV).

Weight (Kg)	Rifampicin dose (mg)	SUSPEND RIFAMPICIN IN EACH VIAL (600MG) IN 10MLS OF WATER FOR INJECTION	Volume used (mL)	Number of vials used	DILUTE IN 500MLS OF 5% DEXTROSE AND ADMINISTER OVER 120 MINUTES
28.5 – 31.49	600		10	1.0	
31.5 – 34.49	660		11	1.1	
34.5 – 37.49	720		12	1.2	
37.5 – 40.49	780		13	1.3	
40.5 – 43.49	840		14	1.4	
43.5 – 46.49	900		15	1.5	
46.5 – 49.49	960		16	1.6	
49.5 – 52.49	1020		17	1.7	
52.5 – 55.49	1080		18	1.8	
55.5 – 58.49	1140		19	1.9	
58.5 – 61.49	1200		20	2.0	
61.5 – 64.49	1260		21	2.1	
64.5 – 67.49	1320		22	2.2	
67.5 – 70.49	1380		23	2.3	
70.5 – 73.49	1440		24	2.4	
73.5 – 76.49	1500		25	2.5	
76.5 – 79.49	1560		26	2.6	
79.5 – 82.49	1620		27	2.7	
82.5 – 85.49	1680		28	2.8	
85.5 – 88.49	1740		29	2.9	
88.5 – 91.49	1800		30	3.0	

**Table 2.** Daily number of tablets of isoniazin, pyrazinamide and ethambutol by weight during the first 14 days for the intravenous rifampicin study arm (arm R20IV).

Baseline weight (kg)	ISONIAZID 100 mg*	PYRAZANAMIDE 500 mg*	ETHAMBUTOL 400 mg
≤30–33	1.5	1.5	1
34	1.5	1.5	2
35–44	2	2	2
45–54	2.5	2.5	2
55	3	3	2
56–64	3	3	3
65	3	3.5	3
≥66	3.5	3.5	3

After 14 days of intravenous rifampicin the participant will be switched to high dose oral rifampicin (35mg/kg) for the remaining 6 weeks of the intervention period administered as per the participants in the high dose oral rifampicin arm.

**High dose oral rifampicin arm (R35PO)**

Oral rifampicin at 25mg/kg for 8 weeks, in addition to standard fixed dose combination quadruple antituberculous tablets containing ~10mg/kg of rifampicin, will be administered for the first 8 weeks. Fixed-dose combination tablets according to weight bands as presented ( Table 3) will be dispensed with additional 300mg oral rifampicin tablets to make the rifampicin dose up to ~35mg/kg/day. All study drugs will be taken orally during this period (either as intact tablets or via nasogastric tube). Study drugs will be administered under directly observed therapy by nursing staff whilst the participant remains in hospital. After discharge study drugs will be dispensed to participants at the week 4 visit. Fixed dose combination antituberculous therapy will be prescribed through routine care pathways initially via the hospital and subsequently via linked TB clinics according to National Guidelines. Participants will be weighed at each visit and TB drug dose will be adjusted as necessary. At the end of the intervention period (week 8) the participant begins standard of care continuation phase TB treatment ( Table 4).

**Table 3.** Daily administration of oral rifampicin during the first 8 weeks in the high dose oral rifampicin arm (R35PO) and week 2–8 in arm R20IV.

Weight	Number of RHZE tabs (150/75/400/275 mg)	Additional rifampicin 300mg tablets	Total rifampicin dose (mg)
30–37 kg	2 tabs	3 tabs	1200
38–54 kg	3 tabs	4 tabs	1650
55–70 kg	4 tabs	5 tabs	2100
≥ 71 kg	5 tabs	6 tabs	2550

**Table 4.** Daily number of fixed dose tablet in the control arm (arm R10PO) during week 0–8 and for all arms during the continuation phase of treatment.

Weight	Intensive Phase (week 0–8)	Continuation Phase (month 3–12)	
	RHZE fixed dose combination daily (150/75/400/275 mg) ^3^	RH (150/75) ^3^	RH (300/150) ^3^
30–37 kg	2 tabs	2 tabs	
38–54 kg	3 tabs	3 tabs	
55–70 kg	4 tabs		2 tabs
≥ 71 kg	5 tabs		2 tabs

**Control arm, standard of care arm (R10PO)**

The control arm will receive standard of care TB treatment: Oral rifampicin ~10 mg/kg, isoniazid ~5 mg/kg, pyrazinamide ~25 mg/kg, ethambutol ~20 mg/kg in fixed-dose combination tablets according to weight bands for 8 weeks ( Table 4). Participants will receive intact tablets or via nasogastric tube. Fixed dose combination antituberculous therapy will be prescribed through routine care pathways initially via the hospital and subsequently via at TB clinics according to National Guidelines.

***Pharmacokinetic sampling.*** Intensive plasma PK sampling will take place on day 2(+/-1) at hours 0, 2, 4 and 8 post dose as well as a single CSF sample. Where possible a single plasma and CSF sample will be collected on day 14 (+/- 2 days).

***Blood test monitoring.*** Routine blood monitoring will take place on days 1, 3, 7 and 14, week 4 and week 8 to monitor for any rifampicin-related toxicity ( Table 5 and Table 6).

**Table 5.** In-patient schedule of events.

Study visit	Screening	Enrolment D1	Day 2	Day 3	Day 7	Day 10	Day 14	Further Hospitalization
Visit window (days)			±1	±1	±1	±1	±2	
Screening Consent	X							
Assess eligibility criteria		X						
Informed enrolment consent		X						
Clinical history and examination								
Past medical history	(X)							
Medication review	(X)	X	X	X	X	X	X	X
Document HIV status	(X)							
Current symptoms	(X)	X	X	X	X	X	X	X
Examination	(X)	X	X	X	X	X	X	X
GCS score	(X)	X	X	X	X	X	X	X
BMRC disease grade		X	X	X	X	X	X	X
Adverse events assessment ^b^		X	X	X	X	X	X	X
Investigations								
HIV-test (if not known positive)	(X)							
Cryptococcal antigen	(X)							
Sodium/Potassium	X ^i^	X ^i^			X		X	X
Glucose (bedside)	(X)							
Creatinine ^h^	X ^i^	X ^i^			X		X	X
Hepatic panel ^a^	X ^i^	X ^i^		X	X		X	X
Blood Count (including differential)	X ^i^	X ^i^			X			X
CD4 count if HIV-positive	X ^i^	X ^i^						
Pregnancy test	Women							
CSF sample (aim >10ml, as per SOP ^c^)	(X)		X ^d^	as clinically indicated (storage of remaining specimen)			X (Paired plasma)	X (as clinically indicated)
Plasma PK sampling 0, 2, 4, 8 hrs			X					
Sparse plasma PK (one sample)							X	
Chest radiograph	(+/-)							
CT head ^f^	+/-	+/-						
Abdominal Ultrasound Scan		X						
Urine sample ^g^	(X)							
Blood/DNA/RNA storage		with consent						with consent
Approx. volume blood (mL)	0	20	20	3	7	0	7	7

**()**= part of routine medical care **^a^ Hepatic panel** = alanine aminotransferase (ALT), alkaline phosphatase (ALP) and bilirubin. Hepatitis BsAg, Hepatitis C Ab and INR will be added if baseline ALT is elevated. In the event of DILI hepatic panel will be performed more regularly. **^b^ Adverse events** will be recorded according to DAIDS toxicity scale **^c^ SOP** = standard operating procedure detailing exact processing and testing algorithm of CSF **^d^ CSF PK** will be timing randomised to early (2–4hrs) or medium (4–6hrs) or late interval (6–8hrs) post dose. **^f^ Contrast enhanced CT head** is indicated if there is focal neurology at baseline or during enrolment period ^g^ **Urine** sample may be collected for testing with LAM (lipoarobinmannan) or urine Xpert MTB/Rif Ultra as part of TB work-up ^h^ Additional **renal monitoring** will be undertaken in those with abnormal baseline creatinine ^i^ **Baseline bloods** must occur at either screening or enrolment visit. It is likely these visits will be on the same day. If baseline bloods were done at screening and enrolment occurs >48 hours later baseline blood tests will be repeated.

**Table 6.** Out-patient schedule of events.

Study visit	Week 4	Week 8	Week 12	Week 18	Week 24	Sick Visit
Visit window (weeks)	±1	±1	±1	±2	±2	As needed
Dispensing of study drug		X	standard fixed dose therapy til 12 months as per local guidelines			
Interim history	X	X	X	TELEPHONE CONSULTATION OR IN PERSON	X	X
Adverse events assessment	X	X	X		X	X
Medication review	X	X	X		X	X
Examination	X	X	X		X	X
Modified Rankin score		X			X	
Detailed neurocognitive follow-up		X			X	
Center for Epidemiological Studies of Depression Scale (CES-D)		X			X	
Sodium, potassium						X (if clinically indicated)
Creatinine						X (if clinically indicated)
Hepatic Panel ^a^	X	X				X (if clinically indicated)
FBC, differential ^b^						X (if clinically indicated)
Storage bloods ^c^		X				X (IRIS event only)
CSF with storage						X (if clinically indicated)
ART counselling ^d^	X	X				
Commence/switch ART ^d^		X				
Total volume of blood (ml)		20				20

^a^Hepatic panel = ALT, ALP, bilirubin +/- INR

^b^FBC; full/complete blood count

^c^Blood will be collected and stored for research if storage consent has been received, refer to site SOP.

^d^HIV-infected patients not receiving effective ART, ART counselling to be performed by qualified counsellors as per standard clinic procedures. Physician discretion allowed for timing of ART initiation.

***Neurocognitive assessment.*** Where possible a detailed neurocognitive assessment will take place on week 8 and week 24.

***Antiretroviral therapy.*** HIV-positive participants who are ART naïve or who have defaulted ART will initiate ART after completion of the intensive phase of TB treatment (at week 8) in accordance with WHO and Ugandan guidelines. An efavirenz-based regimen or dose-adjusted (twice daily) dolutegravir-based regimen would be used in accordance with Ugandan HIV treatment guidelines (currently under revision) ^32^. Participants who were enrolled on a failing efavirenz-based regimen will be switched at week 8 to a dose-adjusted dolutegravir-based regimen. Current protease inhibitor or nevirapine-based ART is an exclusion criteria so we do not anticipate having participants on these agents. All participants will be registered at the Infectious Diseases Institute HIV clinic or the Mbarara Hospital HIV clinic such that their HIV care lies within the national HIV service framework.

### Adverse events and safety reporting

The principles of ICH GCP require that both investigators and sponsors follow specific procedures when notifying adverse events or reactions in clinical trials. The definitions of the EU Directive 2001/20/EC Article 2 based on the principles of ICH GCP apply to this trial protocol. All adverse events will be assessed for seriousness, causality and expectedness. Causality in relation to study drug (rifampicin) is assessed as unrelated, unlikely, possible, probable or definite based on temporal relationship and clinical judgement. If the event is serious and unrelated or unlikely to be related it is classified as an SAE. If the event is possibly, probably or definitely related it is classified as a Serious Adverse Reaction (SAR). Expectedness of the adverse reaction is assessed using the summary of product characteristics (SPC) at the time of the event. An unexpected adverse reaction is one not previously reported in the SPC, or one that is more frequent or more severe than previously reported. If a SAR is assessed as being unexpected it becomes a suspected unexpected serious adverse reaction (SUSAR). Intensity will be graded using the DAIDS toxicity scale.

The Trial Management Group at the Infectious Diseases Institute (IDI) must be notified of all grade 3-5 AEs, SAEs and SARs within 24 hours, thereafter they are responsible for reporting SAEs, SUSARs to the Sponsor, regulatory authorities and ethics committees in accordance with local regulatory and institutional guidelines.

***Interim analyses.*** Interim analysis of safety will occur after 24 participants (8 per arm) have completed the week 8 visit. The committee can modify the frequency of interim analysis and early termination could occur if the data safety committee decides there is an unacceptable level of toxicity in any of the investigational arms.

### Data collection

***Baseline and subsequent assessment.*** Participants will be followed as in-patients for the first 14 days and then approximately 4 weekly as outpatients until week 24, see Table 5 and Table 6. If a trial participant dies during the in-patient period additional consent will be sought from family members for a post-mortem examination to explore cause of death and levels of rifampicin in brain tissue. Recruitment will continue for 12–18 months with an additional 6 months follow-up period. The trial will be considered closed when the last participant has completed 24 weeks in the study, all SAEs resolved and all follow-up and laboratory reports have been received.

***Data handling and data management.*** Source documents are made up of detailed case report forms (CRFs), laboratory results, radiology results and other relevant documents. Data entry will occur via the DataFax system, whereby the paper-based case record forms are scanned, emailed to a server, and data entered by intelligent character recognition. After an initial automated error-checking, secondary review for accuracy is then performed by the DataFax team at the Infectious Diseases Institute, Uganda. The DataFax system allows for automated data queries to alert for any missing data on an ongoing basis. Second, this also allows for permanent archiving and potential remote review by oversight bodies. Study forms will be harmonized between all study sites enabling multi-site data management. The investigator will retain study essential source documents for 20-years after the completion of the study, as per Ugandan guidelines. Digital images of the source documents will be retained for an indefinite period.

***Quality control and assurance.*** Site monitoring is conducted to ensure that the human subject protection, study procedures, laboratory, study intervention administration, and data collection processes are of high quality and meet the sponsor, ICH E6 and regulatory guidelines. The study may be subject to audit by the London School of Hygiene & Tropical Medicine under their remit as Sponsor, as well as other regulatory bodies to ensure adherence to Good Clinical Practice.

### Statistical considerations

***Sample size.*** Approximately 800 adults presenting with symptoms of meningitis will be assessed, of which it is anticipated two-thirds will have cryptococcal meningitis. The circa 240 patients with negative CSF cryptococcal antigen (crAg) will undergo a comprehensive TBM diagnostic panel (Xpert MTB/Rif, Xpert Ultra, mycobacterial growth indicator tube (MGIT) culture, and/or next generation sequencing) from which we anticipate 60 people with suspected or confirmed TBM will be recruited into the randomised trial.

The sample size is determined following the assumption that PK parameters are normally distributed on the log scale. Estimated rifampicin log-transformed C _max_ standard deviation is taken from *Ruslami et al*. ^1^ The global study significance level (alpha) will be 10%, which is appropriate for phase II studies. Using the Bonferonni correction for multiple testing, each individual comparison will be made with alpha of 5%. Group sample sizes of n=15 achieve 90% power to reject the null hypothesis of equal means when the log-transformed population mean difference is 0.54mg/L with standard deviation of ±0.36mg/L in the control group and ±0.48mg/L (33% higher) in the experimental group, with a significance level of 0.05 using a two-sided, two sample unequal variance t-test. If the standard deviation differs from our estimate, the overall detectable effect size at 90% power will be 1.5 times the standard deviation, and 1.27 times the standard deviation at 80% power. We will not formally compare equivalence of the two investigational arms as this would require a larger sample size. Comparison of the two investigational arms will be primarily descriptive.

This phase II study would be underpowered to detect a survival benefit unless the effect size is large. A one-sided log rank test with 20 subjects per arm provides 80% power at 0.05 significance to detect a hazard ratio of 0.285 when 40% of controls survive 8-weeks. This assumes no more than 1 person drops out or is lost to follow-up per arm. The prior lost to follow up rate has been <1% in recent Ugandan meningitis trials.

***Statistical methods.*** After data cleaning, analysis will proceed according to the pre-specified analysis plan using Stata version 13. The trial will be reported in accordance with CONSORT guidelines and primary analyses will be conducted by the participant’s originally assigned group (ITT). Analyses will be conducted on individuals from both study sites together and all analyses will be adjusted for site, BMRC grade and any baseline variables associated with missingness of the analysis outcome.

***Primary analysis***

**Pharmacokinetic analysis**

The following pharmacokinetic parameters will be calculated using standard two-stage approach involving calculation of pharmacokinetic parameters in those patients in whom a full PK curve was recorded:

1. C _max_ - the peak concentration of the drug after administration
2. T _max_ - the time to reach C _max_
3. AUC _0-24_ - area under the time concentration curve from hour 0 to hour 24
4. Time > MIC is the duration of time where the drug concentration level is greater than the minimal inhibitory concentration for the *M. tuberculosis* isolate (either the individual or the median MIC of the study population)

**Safety analysis**

The number and proportion of individuals who reported any kind of clinical grade 3-5 AE during the study period will be presented by intervention arm. Data for safety will be analysed in accordance with the ITT principle. The main analysis will be a composite safety endpoint over the 8-week intervention period:

- AEs, clinical Grade 3-5 as classified by DAIDS Toxicity Scale
- All SAEs
- DILI, grade 3-5 (ALT >3x ULN with symptoms or 5x ULN without symptoms)
- Discontinuation of rifampicin for >5 days in the first 8 weeks for any cause

Time to experiencing an adverse event will be compared between treatment arms using cumulative incidence functions and a Cox proportional hazards regression model adjusted for site and BMRC TBM grade. Death will be considered a competing risk. The individual subgroups of AEs that make up the composite endpoint will be summarised. A secondary analysis of rifampicin-related toxicity (probable or definitely related, as assigned on the adverse events case record form) will be presented by intervention arm.

**Clinical outcomes**

Survival at 8 and 24 weeks will be calculated using the risk difference, from a generalised linear regression model adjusted for site and BMRC TBM grade. Kaplan-Meier curves will also be used to compare time to death between treatment arms, with censoring and loss to follow-up handled as previously described. Cox proportion hazards regression will be used to assess the effects of rifampicin dose, plasma/CSF rifampicin concentration, HIV, MRC severity grade and baseline GCS on survival.

When calculating time to normalization of GCS continuous-valued secondary endpoints will be compared with general linear models or Wilcoxon rank-sum tests as appropriate. Death can be considered a competing risk. χ ^2^ test will be used for comparison of proportions of patients with TBM-IRIS by intervention arm.

***Secondary analysis.*** We will explore the relationship between rifampicin exposures and clinical outcomes using a population pharmacokinetic-pharmacodynamic model. We will consider C _max_ and AUC _0-24h_ as the exposure variables. Survival, proportion with normalisation of conscious level and functional status (as determined by modified Rankin score) are the response variables.

***Planned subgroup analyses.*** Specified high-risk subgroups are of interest because of potential different responses to intravenous rifampicin from both an efficacy and safety perspective, and the clinical utility in future physician decision-making. With standard therapy, worse clinical outcome is associated with altered mental status, high organism burden. Subgroups of interest include:

1. TBM Diagnostic category (Definite, Probable, Possible) ^33^
2. British Medical Research Council (BMRC) TBM disease severity ^7^
3. CSF inflammation (CSF white blood cells) by tertiles
4. CD4 at study entry
5. ART status at study entry

***Analysis of ancillary studies.*** The results of ancillary studies will be reported separately from the main trial.

Findings of the diagnostic sub-study will be reported in line with the Standards for the Reporting of Diagnostic Accuracy studies (STARD) guidelines. Sensitivity and specificity will be calculated against a composite reference standard (any positive CSF test for *M. tuberculosis*) and against the published uniform case definition of ‘probable’ or ‘definite’ TBM ^33^. A latent class analysis will also be performed.

The studies on brain tissue concentration at autopsy will be primarily descriptive.

### Ethical considerations

***Confidentiality.*** Participants will be identified only by means of a coded identification number specific to each participant. All participant-related information (including CRFs, laboratory results, radiology reports etc.) will be kept strictly confidential. All records will be kept in a secure, locked location and only research staff will have access to the records. With consent, information relevant for the future HIV care will be shared with relevant HIV clinic for continuity of care and patient safety.

All computerized databases will identify participants by numeric codes only, and will be password-protected. Upon request, participant records will be made available to the study sponsor, monitors, applicable regulatory entities, including the Uganda National Council of Science and Technology, Uganda National Drug Authority, Mulago Institutional Review Board, or London School of Hygiene and Tropical Medicine.

***Consent.*** An estimated >80% of the study population will have altered mental status at initial hospital presentation. Those presenting with meningitis and altered mental status are at the greatest risk of death, and enhanced TBM therapy may have the greatest benefit for them. This hypothesis deserves testing via inclusion into the clinical trial. Secondly, as most persons with TBM present with altered mental status, in order to make results generalizable to all TBM patients, we wish to offer enrolment to all persons. Subjects unable to give informed consent due to altered mental status may have surrogate consent provided by proxy from their caregiver/next of kin.

For subjects enrolled by surrogate consent, if and when the participant regains the physical and mental capacity to give consent, information will be provided to them and written informed consent will be sought for continuation in the trial. If a patient or representative declines to give consent for continuation at this stage, his/her wishes will be respected.

A person who speaks and understands the language of the informed consent document, but does not read and write, can be enrolled in a study by "making their mark" or via a thumbprint on the informed consent document. In this event, an impartial, literate third party must witness the entire consent process and sign the informed consent document. The witness’s name, signature, and relationship must be recorded on the informed consent document. A member of the study team is not an impartial third party.

Supplementary File 1 contains the participant information sheet and consent form.

***Sample use and storage.*** Additional consent will be sought for the long-term storage of samples (including blood, spinal fluid, DNA/RNA) for use in future research. In this case, samples will be stored for the purposes of future research, unless the subject asks for them to be destroyed. Alternatively, subjects may give limited consent to the collection and testing of samples for the purposes of this research only, after which the samples will be destroyed.

***Withdrawals.*** Subjects may withdraw from the study at any time by withdrawing subject consent, and will be eligible to continue to receive TB treatment from a primary TB clinic of their choice. Subjects enrolled in the study but choosing to leave the hospital early against medical advice will continue to participate in the study if they wish. Additional phone calls by study personnel will encourage the subject to seek follow up TB care and to re-join the trial per the on-going schedule of events. Assessment of vital status will continue via telephone calls at a minimum, unless consent is completely withdrawn. Participants will be asked if they would like their accrued data to be destroyed.

***Ethical approval.*** The investigators have obtained approval from the Research Ethics Committees of the London School of Hygiene and Tropical Medicine (14388), as well as the Mulago Hospital Institutional Review Board (MHREC1260), the Uganda National Council of Science and Technology, the Ugandan National Drug Authority and Mbarara Hospital.

Any further amendments will be submitted and approved by each ethics committee.

### Trial committees

The trial sponsor is London School of Hygiene and Tropical Medicine (LSHTM; Keppel Street, London, WC1E 7HT, UK; Tel: 0207 6368636). The trial management group (TMG) will oversee day to day management of the trial and is formed of the principal investigators (PI), site PI in Mbarara, a neurologist and a statistical advisor. The TMG will meet weekly. The trial steering committee (TSC) has members of the TMG and independent members (Professor Guy Thwaites (Chair), Professor Alison Elliott, Professor Reinout van Crevel, Dr Joe Jarvis, Dr Frank Mugabe (Head of National TB and Leprosy Programme), a patient representative). The TSC provides supervision for the trial and advice through the independent Chair. The data safety committee (DSC) (Professor Robert Wilkinson (Chair), Dr William Worodria, Dr Mindy Clarke, Dr Agnes Kiragga (independent statistician), Dr Christine Sekaggya-Wiltshire) will advise the TSC regarding continuation, modification or premature closure of the trial. The DMC is independent from the Sponsor.

### Publication policy

We will share results though presentations at scientific conferences and in peer-reviewed open-access journals. De-identified individual patient data will be stored on LSHTM secure data repository (LSHTM Data Compass) for patients who have given consent to data sharing at the time of enrolment.

## Discussion

Tuberculous meningitis is highly fatal despite current WHO recommended therapy, particularly in HIV-positive people, who are 2–3 times more likely to die than their HIV-negative counterparts. Recent evidence from Indonesian phase II studies suggests that significantly higher dose IV or oral rifampicin enhances CNS penetration and may reduce mortality. However, there is currently no available pharmacokinetic data on high-dose rifampicin for TBM in an HIV-positive African population. Furthermore, intravenous rifampicin is not widely available so finding a bioequivalent oral dose, that reaches target levels in the CNS, is a priority.

In this phase II study we anticipate the sample size will not be sufficient to detect small differences in mortality between the treatment arms. However, the pharmacokinetic and pharmacodynamic data will be used to define optimal rifampicin dose, and route of administration in an HIV-positive population, and this will be taken forward into a larger phase III randomised controlled trial, if safe and tolerable.

## Data availability

No data are associated with this article.

## Supplementary material

Supplementary File 1: Participant information sheet and consent form.
