# Supplementary material for: High dose oral and intravenous rifampicin for improved survival from adult tuberculous meningitis: a phase II open-label randomised controlled trial (the RifT study)
Source: Wellcome Open Res. 2018 Jul 10;3:83. [Version 1] doi: 10.12688/wellcomeopenres.14691.1 (PMC6113880; doi:10.12688/wellcomeopenres.14691.1)
Supplement: Supplementary file 1 [file wellcomeopenres-3-15997-s0000.tgz › 7f54bbdb-3333-4265-9822-cf081f5b2184.pdf]

PARTICIPANT INFORMATION SHEET FOR ENROLLMENT

STUDY: High Dose Intravenous and Oral Rifampicin to Improve Survival of Adult Tuberculous meningitis: a Phase II Open Label Randomized Controlled Trial (The RIFT trial)
PRINICPAL INVESTIGATOR: Dr Fiona Cresswell and Dr David Meya
SPONSOR: London School of Hygiene and Tropical Medicine, UK

1. Why am I being asked to volunteer?

You are being invited to take part in a research study. Your participation is voluntary which means you can choose whether or not to participate. Please take time to consider the following information before deciding whether you wish to participate in this study. We will explain this carefully but please feel free to ask questions.

2. What is the purpose of the study?

Tuberculous (TB) meningitis is a life threatening infection around the brain. Fortunately, this infection is treatable but even with standard treatment some people are left with disability or may die. For this reason, researchers have been searching for better ways to treat this infection. There are four standard TB medicines: rifampicin, isoniazid, pyrazinamide and ethambutol, Researchers have recently discovered that giving a higher dose of rifampicin through an intravenous (IV) injection into a vein or by mouth in tablet form can increase the amount of rifampicin getting to the brain. Whether this will improve survival and reduce disability from TBM is not known.

The purpose of this study is to determine whether rifampicin when given higher than normal dose intravenously or orally will be more effective than usual dose of oral rifampicin for treating TB meningitis.

3. Why have I been chosen?

You have been chosen because we suspect that you have TB meningitis. We hope to enrol 60 people with TB meningitis into this study in Uganda.

4. Do I have to take part?

No, it is up to you to decide to join the study. If you agree to take part, we will then ask you to sign a consent form. You are free to withdraw at any time, without giving a reason.

5. What will happen to me if I take part?

We will discuss the treatment, procedures and follow-up involved in the study in detail.

a. Treatment

You will be allocated to receive either:

1. A higher dose rifampicin of 20mg/kg by intravenous injection once daily for 14 days, followed by higher dose of rifampicin of 35mg/kg taken as tablets for 6 weeks; **OR**
2. A higher dose rifampicin of 35mg/kg taken as tablets for 8 weeks; **OR**
3. Usual dose rifampicin of 10mg/kg once per day given as tablets.

Whether you receive the higher dose of rifampicin will be selected by chance (such as flipping a coin). During this 8-week period you will receive the usual doses of the other 3 standard TB medicines.
After 8 weeks, you will receive the usual two medicines (rifampicin and isoniazid as combined tablets) for 10 more months, as per treatment guidelines for TB meningitis in Uganda. It is very important that you complete all 12 months of treatment so the infection does not come back. You will be required to take all your medications faithfully. It is unknown for sure whether a higher than normal dose rifampicin will improve treatment of your infection.

b. Procedures

The doctors have taken a sample of the fluid surrounding your spinal cord through a needle in your lower back, this is called a lumbar puncture (LP). If you are agreeable, we ask to repeat this LP twice (on day 2 and again on day 12) to measure the amount of drug reaching your spinal fluid and to see whether the infection around your brain is improving. This is a research test, which

would not occur if you decide not to enrol in this study. You will also have some blood samples taken today and several other times over the next 2 weeks. You will receive more intensive monitoring of your medical care by the study doctors. This may include ultrasound scans or brain scans as needed.

c. Follow-up

As TB meningitis is a very serious condition we advise you to remain in hospital for the next 14 days for monitoring and to receive medications and fluid through a drip. We will monitor your liver blood tests and may rarely need to stop or modify the TB treatment if your liver is inflamed. After leaving the hospital, you will be required to return for clinic visits at 4 weeks, 8 weeks, 16 weeks and 24 weeks after beginning of TB treatment. During the follow-up appointments you will meet with a study doctor and nurse who will check on your wellbeing and discuss medications with you. At the week 8 and week 24 visit a nurse will help you to complete detailed questionnaires to measure your brain function and mood. The total duration of the study is 24 weeks. If you are not already on anti-retroviral therapy (ART) this will be discussed with you, and started in accordance with national guidelines. If you are unable to travel to attend clinic, study staff may come to your home with your permission. We also request for your permission to be contacted by phone by the study staff to check on your well being if they have any concerns.

6. Expenses and Payment

You will not be paid for participating in this study, but you will be given money to refund your transport expenses, time and inconvenience up to 30,000 Ugandan Shillings per study related visit in the clinic.

7. What are the risks and disadvantages of taking part?

- Until this study is completed, there is no way of knowing for sure if higher dose rifampicin is helpful for treating TB meningitis. For this reason, there is a risk that you could be receiving more medicine that is not beneficial.
- It is possible that higher dose rifampicin may be more toxic. Rifampicin causes harmless red discolouration of the bodily fluids (e.g. urine), can cause rash, flu-like symptoms and inflammation of the liver. The side effects are usually mild and go away spontaneously or on stopping the medicine. However, as with any medicine severe adverse reactions can occur very rarely. Small studies of rifampicin at higher doses have shown that it is safe, with no increase in toxicity.
- Risks of lumbar puncture include bleeding around the puncture site, and rarely infection or fluid leak causing a headache. Having an LP has the same risks for you whether you have the LP in this study or in standard clinical care. An LP was required for diagnosis, and it is recommended that you receive at 2 more LPs in approximately 1-2 days and 10-14 days to check if the infection is improving. This is probably more than you would receive during standard hospital care.

8. What are the possible benefits of taking part?

We know from prior research that rifampicin is the most important drug in the treatment of TBM but when given at standard dose very little reaches the spinal fluid, which is the site of the infection. Giving higher doses of rifampicin by mouth or IV allows more drug to reach the spinal fluid. It is possible that higher levels of rifampicin may kill the *M. Tuberculosis* bacteria more effectively. However, whether this will lead onto reduced disability or improved survival is not known. During the trial you will receive more intensive monitoring that you would likely receive in routine TB care and this may mean that complications can be detected and treated earlier.

9. Risk to unborn child

Pregnant or breast-feeding women will be excluded from the study. Rifampicin in standard doses is know to be safe but there is no safety data on higher doses so female study participants are strongly advised *not* to become pregnant during the first 8 weeks of the study. If you are sexually active, we recommend that you use condoms to prevent pregnancy. If you later become pregnant or think you may be pregnant, please notify the study doctor.

10. What if I don't want to participate?

If you decide not to take part you will receive the standard TBM treatment from the hospital medical team. You would still have to have some monitoring blood tests and possibly another lumbar puncture. It would also be necessary to complete 12 months of TB treatment.

11. Will my taking part in the study be kept confidential?

Yes. All information collected about you during the course of the research will be kept strictly confidential. Any information about you that leaves the clinic/hospital will have your name and address removed so that you cannot be recognised from it. Your

records for the study may be reviewed by the University or other government regulatory authorities to assure the accuracy and quality of the records and the correct conduct of the research study.

.

12. What will happen if I don't want to carry on with the study?

Agreeing to participate in this study is voluntary and you can withdraw from the study at any time by telling the study team. If you withdraw from the study, you can continue to receive your standard TB medicines free from the hospital or at the TB clinic. Any stored blood or tissue samples that can still be identified as yours will be destroyed if you wish (please ask the study doctor).

13. What if something goes wrong?

If you have a concern about any aspect of this study, you should ask to speak to the researchers who will do their best to answer your questions. If you remain unhappy and wish to complain formally, you can do this via the Principal Investigator / Co-Investigators or Mulago Hospital Research Ethics Committee, see details below. If you experience harm or injury as a result of taking part in this study, you may be eligible to claim compensation. You will not be giving up any of your legal rights by signing this consent form. If you are harmed due to someone's negligence, then you may have grounds for a legal action.

14. What will happen to the results of the research study?

The results of this study will be used by researchers and doctors to inform what is the best dosage and route of administration of rifampicin. The best rifampicin regimen can then be examined in a larger study, the results of which can be used by policy makers in making national guidelines for the best way to treat TBM. The results will be published in medical journals and presented at conferences but you will not be identified in any reports or publications.

15. What will happen to any samples I give?

The blood samples and spinal fluid taken during your hospital stay will be analysed in Uganda to measure the levels of the rifampicin and other drugs. All samples will labeled with an identification number and there will be no way that you can be identified from these samples.

The option of giving *additional* consent for long-term storage of samples for approved future research will have been discussed with you in the screening process. If you have provided storage consent for future research the samples will be stored in Kampala. If you have declined to give additional storage consent for future research your samples for will be destroyed at the conclusion of this study. Storage consent for future research can be given at any time before the samples are destroyed.

16. Who has approved the study?

The research is being funded by the Wellcome Trust as part of a Clinical PhD Fellowship in Global Health Research. The international sponsor of the study is London School of Hygiene and Tropical Medicine in the United Kingdom, and the local study team is from the Infectious Diseases Institute, Kampala. Ethical approval to conduct this study has been granted by the Research Ethics Committees at Mulago Hospital, Uganda and London School of Hygiene and Tropical Medicine, UK. The Uganda National Council of Science and Technology has also approved the study.

17. Contacts and Questions

You will be given a clinic appointment card with the phone number before you leave hospital. You may ask any questions you have now or contact the below individuals later. If you have any urgent health concerns or return of your headache, you are encouraged to contact:

Dr Fiona Cresswell on telephone number 0793420173
Dr David Meya on telephone number 0772543730

In case of any questions regarding the Welfare and rights of participants, you should contact:
Dr Nakwagala Fredrick Nelson,
Chairman of the Mulago Research Ethics Committee
Telephone number 0772325869

You will be given a copy of the information sheet and a signed consent form to keep.

Thank you for taking the time to read this information sheet.

INFORMED CONSENT FORM FOR ENROLMENT INTO THE RIFT TRIAL

STUDY: High Dose Intravenous and Oral Rifampicin to Improve Survival of Adult Tuberculous meningitis: a Phase II Open Label Randomized Controlled Trial (The RIFT trial)
PRINICPAL INVESTIGATOR: Dr Fiona Cresswell and Dr David Meya
SPONSORS: London School of Hygiene and Tropical Medicine, UK

	Initial or Thumbprint
I have read or had the particicpant information sheet (version……….) about enrollment into the RifT study read to me in full. I have had opportunity to consider the information and to ask questions and have had my questions answered completely.	
I understand that my participation is voluntary and I am free to withdraw at any time, without giving any reason, without my medical care or legal rights being affected.	
I understand that any relevant sections of my medical notes and data collected during the study may be looked at by responsible individuals from the Sponsor or from regulatory authorities where it is relevant to my taking part in this research. I give permission for these individuals to have access to my records.	
I agree to take part in the above study and will comply with the study medication and follow-up as explained in the patient information sheet.	

Name of Participant	Signature / Thumbprint (with witness)	Date
Name of Guardian or Next of Kin *(if required)*	Signature	Date
Name of Person taking consent	Signature	Date
Principal Investigator	Signature	Date

The participant is illiterate and thumbprint used. As a witness, I confirm that all the information about the study was given and the participant consented to taking part.

Name of Impartial Witness *(if participant thumbprint used)*	Signature	Date
