# Supplementary material for: High dose oral and intravenous rifampicin for improved survival from adult tuberculous meningitis: a phase II open-label randomised controlled trial (the RifT study)
Source: Wellcome Open Res. 2018 Jul 10;3:83. [Version 1] doi: 10.12688/wellcomeopenres.14691.1 (PMC6113880; doi:10.12688/wellcomeopenres.14691.1)
Supplement: Peer review history

# Peer review: Referee response for version 1

**DOI:** 10.21956/wellcomeopenres.15997.r33459

**Published:** 2018-08-28

**Authors:** Mosepele Mosepele

**Affiliations:** 1 University of Botswana & Botswana Harvard AIDS Institute Partnership (BHP), Gaborone, Botswana

Dr Cresswell and team are proposing an important, and long overdue Phase II trial, to test the optimal Rifampicin dose for TBM in a population that suffers excess rates of TBM. Background literature is comprehensive. Rationale for proposed work is clearly stated. Choice of study population is appropriate. Many safety measure are in place.

 Comments:

 Main limitation is that the study sample size will allow descriptive comparison of the two investigational arms, given the effort and resources dedicated to the conduct of the study.

 Rationale for 20mg/kg IV versus 35mg/kg PO could be clarified, as in, data on comparability of these doses should be included in the background section. The design implies that these are potentially comparable.

 Given that primary objective includes RIF drug levels, a possible ancillary study would be to collect and store stool as alteration in gut microbial population has been known to affect drug metabolism in the HIV-infected population. Future studies on banked stool samples may aide in additional understanding on between subject differences in RIF levels.

 Under clinical outcome, it would be important to include HIV RNA level (not just HIV disease) given unknown interactions between high doses of RIF and DTG.

 There is no data on interaction between high doses of RIF and Dolutegravir. Please comment on whether patients on Dolutegravir will be included, and what safety measures will be taken. Please provide preliminary information on pharmacokinetic data/rationale for assuming that 50mg DTG is sufficient in the context of higher doses of RIF (20-35mg/kg) as compared to standard doses of RIF (10mg/kg).

 Will ATT be given with pyridoxine in all 3 arms? If so, what dose(s)?

 Will ATT be given with Cotrimoxazole in all 3 arms for active AIDS defining illness? If so, what dose(s)? State rationale either way.

I have read this submission. I believe that I have an appropriate level of expertise to confirm that it is of an acceptable scientific standard.

# Peer review: Referee response for version 1

**DOI:** 10.21956/wellcomeopenres.15997.r33458

**Published:** 2018-07-23

**Authors:** Joseph Donovan

**Affiliations:** 1 Oxford University Clinical Research Unit, Ho Chi Minh City, Vietnam

Dr Cresswell *et al *present their study protocol for a phase II open label, randomised controlled trial of high dose oral and intravenous rifampicin therapy in a predominantly HIV co-infected population with TBM. The study will allocate patients to one of 3 strategies; intravenous 20mg/kg/day rifampicin for 2-weeks followed by oral rifampicin 35mg/kg/day for 6-weeks, oral 35mg/kg/day rifampicin for 8-weeks, or standard of care oral rifampicin (10mg/kg/day) for 8-weeks. Primary outcomes will be pharmacokinetic parameters in plasma and CSF, and safety.  

 This study aims to provide data on an important research topic; namely the optimum dosing of rifampicin in TBM given the drugs poor penetration into CSF. An HIV co-infected population is unique given their worse outcomes, altered anti-tuberculous drug absorption, and the complex drug interactions that occur due to both cytochrome P450 enzyme induction and inhibition with the various drugs used to treat TB and HIV. 

 This protocol is well written, and the design well thought through. The introduction has a logical flow and presents the problem and the available evidence up to this point. Important sub studies will assess GeneXpert Ultra as a diagnostic tool in TBM, and interestingly brain tissue rifampicin concentrations on autopsy specimens, given the importance of rifampicin’s brain tissue penetration as well as its CSF penetration. 

 The study hypotheses and objectives are clearly described, and the methods reproducible. Identifying any safety issues is clearly important, and the data collection timepoints appear logical. Drug induced liver injury (DILI) will be monitored for and ‘participants will be replaced a posteriori if baseline ALT>3x ULN’. Safety analysis will include an analysis of the occurrence of DILI. Efavirenz or dose-adjusted dolutegravir regimens will be used, and patients receiving protease inhibitor or nevirapine-based ART will be excluded, due to their unsuitability with concomitant rifampicin. Study patients requiring changes to pre-established ART during the intervention period (where rifampicin-safe ART becomes unsuitable for any reason) will present a challenge to the team if alternative options become limited, and protease inhibitors must be considered. 

 The authors plan an interim safety analysis after 24 participants (8 per arm) have completed their week 8 visit, to allow modification of the frequency of interim analysis and early study termination if deemed necessary. Given only 60 patients will enrol (20 to each arm), and the mortality in this patient group is expected to be high (many enrolled patients may not survive to their 8-week visit), the authors may find they need to perform this interim analysis sooner, or else perform it when 24 patients (8 in each arm) have been enrolled, and the most recent patient has reached their 8-week follow up or would have reached 8-weeks if they were still alive. Otherwise at the point of the safety analysis the authors may find that too many patients have already been enrolled to make a meaningful change to the study, if indeed any change is required.   

 In summary, this is a clear and well written protocol that addresses an important research question.

I have read this submission. I believe that I have an appropriate level of expertise to confirm that it is of an acceptable scientific standard.
